# Mitochondrial Dysfunction as a Driver of Meta-Inflammation in Aging: The Emerging Role of PDK4 in Bioenergetic Reprogramming and Inflammatory Amplification

**DOI:** 10.3390/cells15151404

**Published:** 2026-08-03

**Authors:** Md Riad Chowdhury, Gui-Hwa Jeong, In-Kyu Lee

**Affiliations:** 1 Department of Biomedical Science, Graduate School, Kyungpook National University, Daegu 41944, Republic of Korea; riadchy01@gmail.com; 2 Department of Internal Medicine, CHA Gumi Medical Center, CHA University, Gumi 39295, Republic of Korea; ghjeong93@chamc.co.kr; 3 Department of Internal Medicine, Kyungpook National University Hospital, School of Medicine, Kyungpook National University, Daegu 41944, Republic of Korea

**Keywords:** mitochondrial dysfunction, meta-inflammation, inflammaging, PDK4, pyruvate dehydrogenase, mtROS, mitophagy, SASP, NLRP3, aging

## Abstract

**Highlights:**

**What are the main findings?**

- Mitochondrial dysfunction acts as a central driver of aging-associated meta-inflammation by promoting mtROS production, mtDNA release, impaired mitophagy, altered NAD^+^ metabolism, and activation of NF-κB, NLRP3, cGAS–STING, and SASP pathways.
- PDK4 emerges as a mitochondrial metabolic checkpoint that restricts pyruvate oxidation, favors lactate accumulation, and may connect altered fuel metabolism to NOX1-derived ROS, SASP activity, and inflammatory amplification.

**What are the implications of the main findings?**

- The PDK4–PDH axis provides a useful framework for understanding how mitochondrial fuel restriction may contribute to chronic inflammation across aging tissues, including skeletal muscle, adipose tissue, brain, and kidney.
- Therapeutic strategies that restore mitochondrial fuel flux, improve mitophagy, regulate redox balance, and normalize maladaptive PDK4 activity may help reduce meta-inflammation and preserve healthspan, although clinical translation requires tissue- and context-specific validation.

**Abstract:**

Aging is accompanied by a progressive decline in mitochondrial quality, bioenergetic flexibility, and stress resilience. Aging mitochondria are increasingly recognized as active inflammatory signaling platforms rather than passive targets of cellular damage. Excess mtROS, leaked mtDNA, defective mitophagy, altered NAD^+^ metabolism, and impaired pyruvate oxidation together create a cellular environment that favors persistent inflammatory activation. These signals engage NF-κB, NLRP3 inflammasome, cGAS–STING, and SASP pathways, allowing mitochondrial stress to spread from organelle dysfunction to tissue-level inflammation. Within this framework, pyruvate dehydrogenase kinase 4 (PDK4) is of particular interest because it directly controls mitochondrial pyruvate entry through inhibition of the pyruvate dehydrogenase complex. By phosphorylating and inhibiting the pyruvate dehydrogenase complex, PDK4 limits mitochondrial pyruvate oxidation and favors lactate accumulation, fatty acid utilization, and redox-inflammatory signaling. Recent work in senescent cells links PDK4-dependent lactate accumulation to NOX1-derived ROS and SASP activity, suggesting a direct route by which altered fuel handling may reinforce inflammation. Here, we review mitochondrial dysfunction as the organizing principle of age-associated meta-inflammation, discuss PDK4 as a central metabolic checkpoint, examine tissue-specific consequences in muscle, adipose tissue, brain, and kidney, and evaluate therapeutic strategies aimed at restoring mitochondrial function to suppress chronic inflammation and preserve healthspan.

## 1. Introduction

Aging is increasingly understood as a systems-level decline in cellular maintenance rather than the isolated failure of a single organelle or pathway [1,2]. The updated hallmarks of aging include mitochondrial dysfunction, disabled macroautophagy, chronic inflammation, deregulated nutrient sensing, altered intercellular communication, cellular senescence, and epigenetic remodeling [1]. These hallmarks do not operate independently. Damaged mitochondria, persistent stress responses, and inflammatory signaling often develop together and intensify one another over time [1,2]. Among these processes, mitochondrial dysfunction occupies a central position because mitochondria simultaneously govern ATP production, redox balance, metabolite availability, innate immune signaling, apoptosis, and adaptive stress responses [3].

Mitochondria are dynamic organelles that coordinate cellular energy production and stress adaptation [4,5]. Through the tricarboxylic acid cycle and oxidative phosphorylation, mitochondria convert nutrient-derived carbon into reducing equivalents and ATP, while also generating metabolites that regulate biosynthesis, epigenetic state, and immune signaling [5,6]. Beyond ATP production, mitochondria buffer intracellular calcium, regulate apoptosis, generate controlled amounts of ROS for signaling, and continuously remodel their network through fusion, fission, biogenesis, and mitophagy [5,7,8,9]. When these processes are disrupted, mitochondria can shift from homeostatic organelles to inflammatory signaling platforms by producing excess mtROS, releasing mtDNA and other danger-associated molecular patterns, and failing to clear damaged mitochondrial fragments [10,11,12,13]. These basic mitochondrial functions provide the biological foundation for understanding how mitochondrial decline contributes to aging-associated inflammation.

Chronic low-grade inflammation, classically described as inflammaging, is strongly associated with frailty, type 2 diabetes, cardiovascular disease, neurodegeneration, chronic kidney disease, sarcopenia, and mortality in older adults [2,14]. When inflammatory tone is generated or sustained by metabolic dysfunction, nutrient excess, lipid overload, or impaired substrate utilization, the process is commonly referred to as meta-inflammation [15]. A mitochondrial perspective helps explain why metabolic stress and inflammation are difficult to separate during aging: aged mitochondria lose respiratory efficiency, accumulate damage, release danger signals, and mismanage fuel flux [3,10]. These changes activate inflammatory transcription factors and innate immune sensors, while inflammatory cytokines further damage mitochondrial function, creating a self-reinforcing cycle [3,4,10].

In this review, we use the term inflammaging to refer to chronic, low-grade inflammatory activation that develops with age. Meta-inflammation, also referred to as metaflammation, describes inflammation that is initiated or sustained by metabolic stress, nutrient excess, lipid overload, or impaired substrate utilization. We use mitochondrial meta-inflammation to describe the subset of this process in which mitochondrial dysfunction, altered fuel metabolism, mtROS, mitochondrial danger signals, and defective mitophagy act as upstream drivers of inflammatory signaling.

Here, we focus on mitochondrial dysfunction as a major source of the inflammatory signals that sustain aging-associated meta-inflammation. Within this framework, PDK4 is not treated simply as a metabolic enzyme, but as a mitochondrial gatekeeper that determines whether pyruvate is oxidized in the tricarboxylic acid cycle or diverted toward lactate and alternative carbon fates. This distinction is central to aging biology. Efficient mitochondrial pyruvate oxidation supports ATP generation, redox homeostasis, and metabolic flexibility; chronic restriction of pyruvate oxidation can promote lactate accumulation, altered NADH/NAD^+^ balance, ROS production, and inflammatory amplification [16]. Thus, the novelty of this review lies not in describing mitochondrial inflammatory pathways individually, but in integrating them with the PDK4–PDH axis as a metabolic control point that may connect restricted pyruvate oxidation, lactate accumulation, redox imbalance, SASP activation, and tissue-level inflammatory propagation during aging.

## 2. Mitochondrial Dysfunction as the Central Driver of Meta-Inflammation

### 2.1. Decline of Mitochondrial Oxidative Phosphorylation in Aging

**Figure 1:**
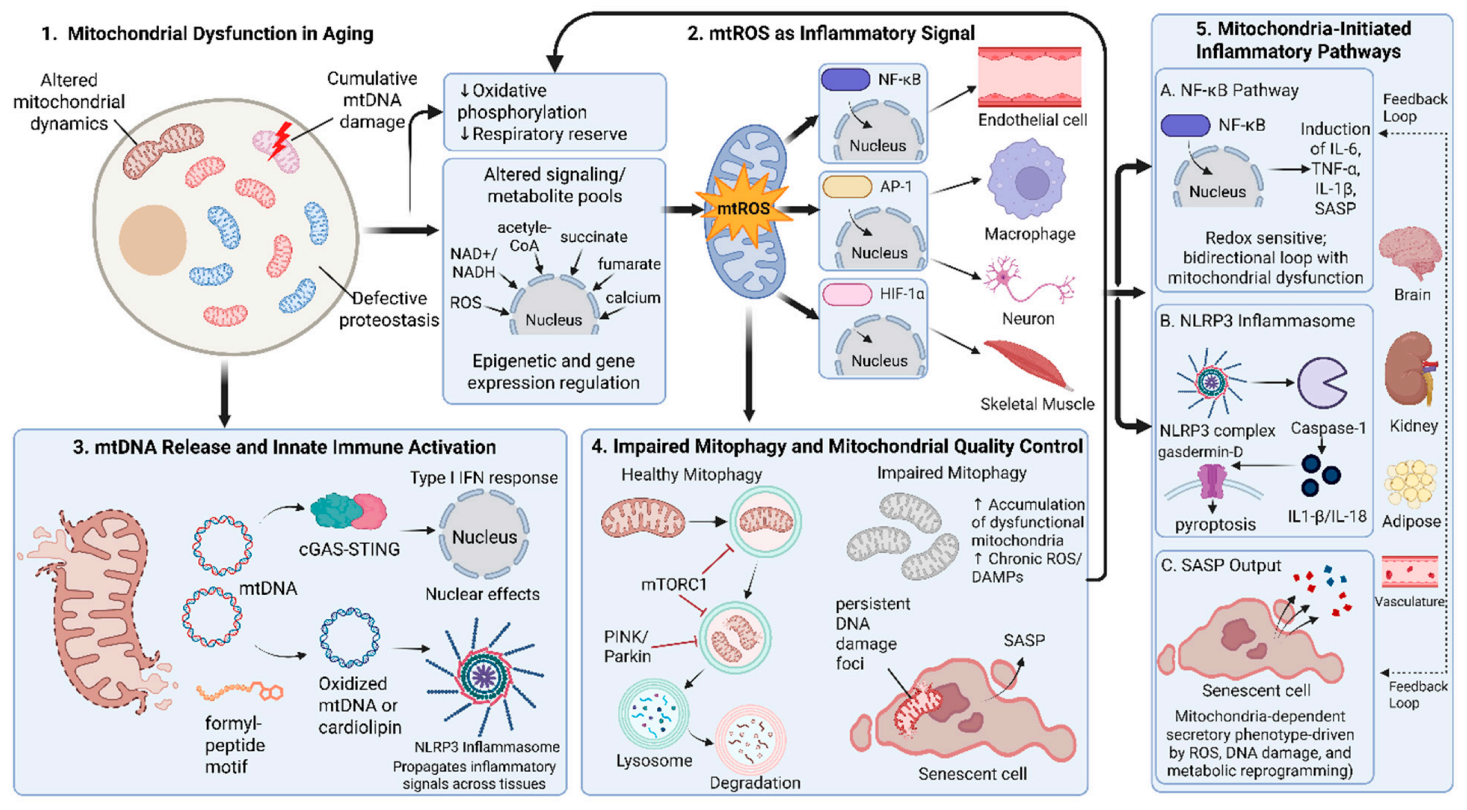
**Mitochondrial dysfunction as a central driver of meta-inflammation in aging.** Aging mitochondria show multiple defects, including impaired oxidative phosphorylation, reduced respiratory reserve, altered dynamics, mtDNA damage, defective proteostasis, and disrupted metabolite/redox balance. These changes increase mitochondrial ROS production, alter NAD^+^/NADH, acetyl-CoA, succinate, fumarate, and calcium signaling, and lower the threshold for inflammatory pathway activation. Damaged mitochondria also release mitochondrial danger signals, including mtDNA and N-formyl peptides, which activate innate immune pathways such as cGAS–STING and NLRP3 inflammasome signaling. In parallel, impaired mitophagy allows dysfunctional mitochondria to persist, sustaining ROS generation, DNA damage responses, and senescence-associated secretory phenotype (SASP) output. Together, mtROS, mtDNA release, defective mitochondrial clearance, NF-κB activation, NLRP3 signaling, and SASP formation sustain chronic meta-inflammation across aging tissues. Arrows indicate directional relationships among mitochondrial damage, redox signaling, innate immune activation, mitophagy failure, and inflammatory output; ROS symbols denote oxidative stress and pathway boxes denote inflammatory signaling modules. Created with BioRender.com (BioRender, Toronto, ON, Canada; https://www.biorender.com/; accessed on 1 July 2026).

Mitochondrial oxidative phosphorylation declines in many aging tissues through cumulative damage to mitochondrial DNA, altered mitochondrial dynamics, reduced biogenesis, impaired electron transport chain activity, and defects in protein import and proteostasis [4,5,17]. These changes reduce respiratory reserve and make cells less able to meet energetic demands during stress [4,5]. However, the inflammatory importance of mitochondrial decline cannot be explained only by lower ATP production. Aged mitochondria also change the intracellular signaling environment by altering NAD^+^/NADH ratios, acetyl-CoA availability, succinate and fumarate pools, calcium handling, and ROS production. These metabolites and redox signals influence transcriptional regulation, histone modification, hypoxia signaling, inflammasome activation, and cytokine production [6] (Figure 1).

Pyruvate oxidation is one of the clearest metabolic points at which mitochondrial function intersects with inflammatory signaling. The pyruvate dehydrogenase complex (PDC) converts pyruvate into acetyl-CoA, thereby linking glycolysis to the TCA cycle [18]. When PDC flux is reduced, pyruvate is diverted toward lactate production or other pathways, and mitochondrial oxidative metabolism becomes less coupled to glucose utilization. This metabolic state resembles aspects of aerobic glycolysis and is frequently observed in inflammatory immune cells, senescent cells, and metabolically stressed tissues [6,16]. In aging, reduced mitochondrial flexibility may therefore help maintain inflammatory reprogramming rather than simply reflect it.

### 2.2. Sources of ROS, Redox Balance, and Inflammatory Signaling in Aging

Reactive oxygen species are generated from multiple cellular sources and should not be viewed as uniformly harmful. Mitochondria produce ROS mainly through electron leakage from the electron transport chain, particularly when respiratory efficiency declines or membrane potential and substrate supply become imbalanced [4,5,19]. NADPH oxidases, including NOX family enzymes, represent another important ROS source and are often activated downstream of cytokines, metabolic stress, and inflammatory receptor signaling [19,20]. Other sources, including peroxisomal metabolism, uncoupled nitric oxide synthase, and endoplasmic reticulum stress, may also contribute to cellular redox imbalance in disease contexts [19].

The biological effect of ROS depends on amount, localization, timing, and antioxidant buffering capacity. At physiological levels, ROS act as signaling molecules involved in hypoxic adaptation, antimicrobial defense, mitohormesis, and exercise-induced mitochondrial remodeling [7,8,21,22]. This is especially important in the context of exercise, where transient ROS generation can activate adaptive pathways that improve mitochondrial biogenesis, antioxidant defense, and metabolic flexibility rather than simply causing oxidative damage [21,22]. Thus, ROS should not be interpreted only as markers of cellular injury.

During aging, however, the balance between ROS production and redox buffering is progressively disturbed. Persistent electron transport chain inefficiency, impaired antioxidant capacity, chronic nutrient stress, inflammatory cytokines, and defective mitophagy can shift ROS from transient adaptive signals to sustained inflammatory triggers [4,5,19,23]. Excess mtROS can activate redox-sensitive pathways including NF-κB, AP-1, and HIF-1α, lower the threshold for NLRP3 inflammasome activation, and amplify SASP-related inflammatory signaling [19,20,23,24]. In this setting, the source of ROS matters: transient exercise-associated ROS may be beneficial, whereas persistent mtROS from damaged mitochondria or chronic NOX-derived ROS may reinforce inflammatory signaling and tissue dysfunction.

This distinction is also relevant to PDK4. Recent evidence suggests that PDK4-dependent lactate accumulation in senescent cells can promote NOX1-derived ROS and support SASP activity [16]. This mechanism differs from ROS generated directly by mitochondrial electron transport, but both sources can converge on redox-sensitive inflammatory pathways. Therefore, aging-associated meta-inflammation should be understood not simply as an increase in total ROS, but as a failure of redox compartmentalization, timing, and resolution.

### 2.3. mtDNA Release and Innate Immune Activation

Mitochondria retain bacterial-like features, including circular DNA and formylated peptides, which can be recognized as danger-associated molecular patterns when released into the cytosol or extracellular space [11,16]. Mitochondrial DNA release can occur after mitochondrial membrane permeabilization, defective mitophagy, lysosomal damage, or severe oxidative stress [11,25]. Cytosolic mtDNA activates cGAS-STING signaling, promoting type I interferon responses, while oxidized mtDNA and mitochondrial cardiolipin have been implicated in NLRP3 inflammasome activation [10,12,26]. These pathways are particularly relevant in aging because mitochondrial damage increases while autophagic clearance declines.

Released mtDNA and inflammatory cytokines can propagate mitochondrial stress signals beyond the initially damaged cell [25]. This may explain how mitochondrial injury in a limited cell population can influence inflammation beyond the original site of damage. From this perspective, meta-inflammation is more than persistent cytokine production; it is partly a form of mitochondrial stress communication between cells and tissues.

### 2.4. Impaired Mitophagy and Mitochondrial Quality Control

Mitophagy, the selective autophagic clearance of damaged mitochondria, is essential for preserving mitochondrial fitness [9,13]. PINK1/Parkin-dependent and receptor-mediated mitophagy pathways remove depolarized or damaged mitochondria before they become sources of excessive ROS and danger signals [9,27]. With aging, autophagy and mitophagy decline through several mechanisms, including mTORC1 hyperactivation, lysosomal dysfunction, reduced autophagosome-lysosome flux, altered NAD^+^/sirtuin signaling, and accumulation of protein aggregates [13]. As a result, damaged mitochondria remain in the cell long enough to sustain ROS production and danger-signal release.

Mitophagy failure is particularly important because it connects mitochondrial damage with cellular senescence. Senescent cells often display enlarged or dysfunctional mitochondrial networks, altered NAD^+^ metabolism, increased ROS production, and a metabolically demanding SASP [28]. Defective removal of damaged mitochondria can maintain DNA damage responses and inflammatory secretory phenotypes. Conversely, interventions that enhance autophagy or mitophagy can reduce inflammatory biomarkers in preclinical models and improve mitochondrial health markers in human studies, suggesting that mitochondrial quality control is a realistic therapeutic axis [29].

## 3. PDK4 as a Mitochondrial Metabolic Checkpoint

### 3.1. PDK4 Regulation of PDH and Mitochondrial Pyruvate Oxidation

**Figure 2:**
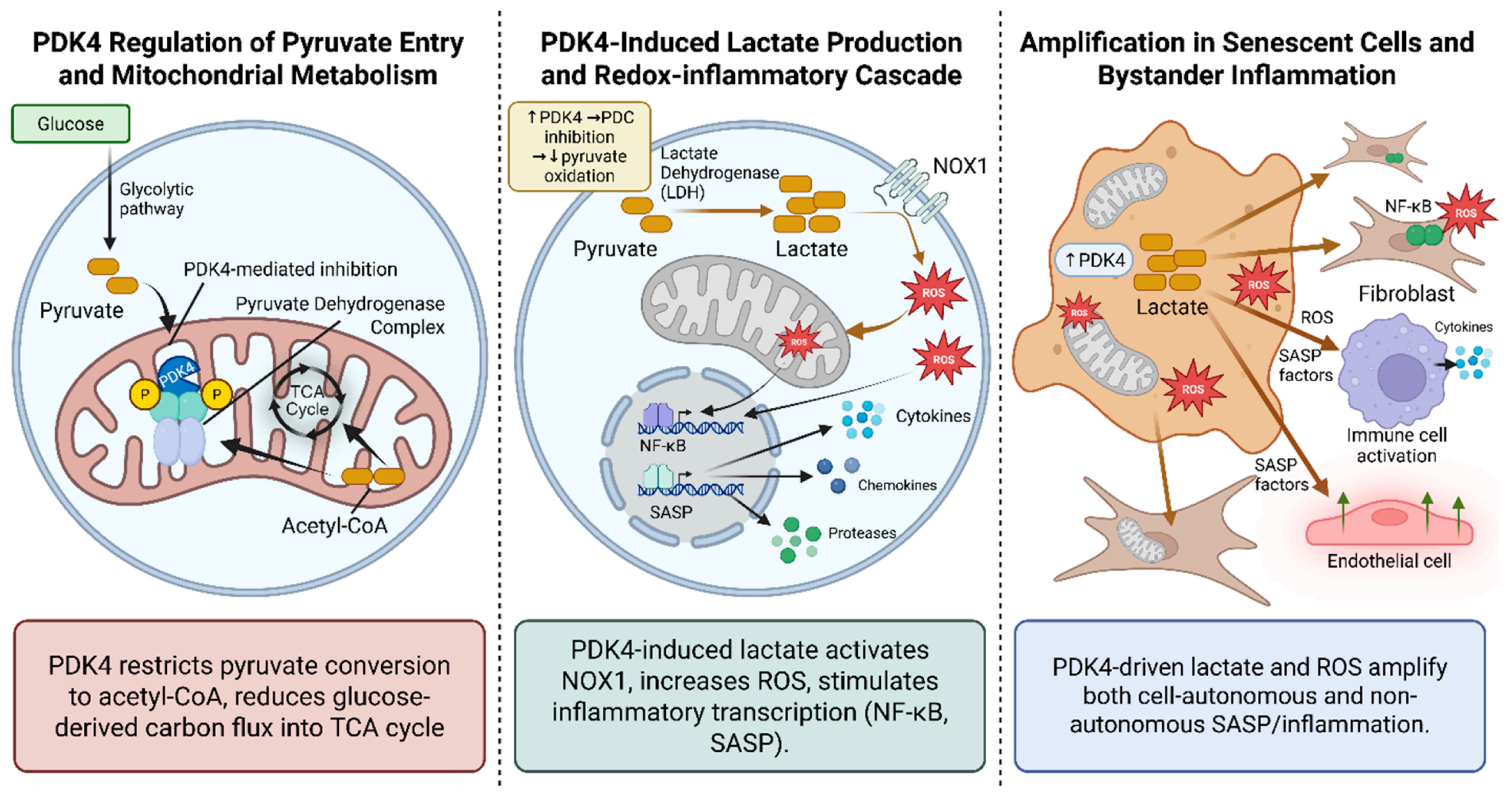
**PDK4 as a mitochondrial metabolic checkpoint linking pyruvate restriction to senescence-associated inflammation.** PDK4 regulates mitochondrial fuel entry by phosphorylating and inhibiting the pyruvate dehydrogenase complex (PDC), thereby reducing conversion of pyruvate into acetyl-CoA and limiting glucose-derived carbon entry into the TCA cycle. When PDK4 activity is transiently increased during fasting, exercise, or fatty acid availability, this response supports physiological substrate switching. However, sustained PDK4 activation during aging, senescence, insulin resistance, or tissue stress may restrict mitochondrial pyruvate oxidation and promote lactate accumulation. Lactate can act as a signaling metabolite that enhances NOX1-dependent ROS production, leading to redox-sensitive activation of NF-κB and inflammatory transcriptional programs. In senescent cells, this PDK4-associated lactate–NOX1–ROS axis may support SASP production and amplify both cell-autonomous and non-cell-autonomous inflammatory signaling in neighboring fibroblasts, endothelial cells, immune cells, and other tissue-resident cells. Thus, PDK4 may link restricted mitochondrial pyruvate oxidation to redox imbalance, SASP activity, and inflammatory spread. Arrows indicate the proposed sequence from PDK4-mediated PDC inhibition to reduced pyruvate oxidation, lactate accumulation, NOX1-derived ROS, NF-κB/SASP activation, and bystander inflammatory signaling. Created with BioRender.com (BioRender, Toronto, ON, Canada; https://www.biorender.com/; accessed on 1 July 2026).

PDK4 is one of four pyruvate dehydrogenase kinase isoforms that phosphorylate and inhibit PDC [18]. By restricting PDC activity, PDK4 limits the conversion of pyruvate into acetyl-CoA and reduces glucose-derived carbon entry into the TCA cycle [18] (Figure 2). Physiologically, PDK4 is induced during fasting, prolonged exercise, glucocorticoid exposure, and increased fatty acid availability, where it contributes to substrate switching by sparing glucose and favoring fatty acid oxidation [30,31,32]. This adaptive response becomes problematic when PDK4 remains chronically elevated in contexts of aging, insulin resistance, senescence, or tissue stress.

The central effect of PDK4 is to limit mitochondrial access to pyruvate. Therefore, its inflammatory relevance cannot be understood from glycolysis alone. When PDK4 activity is high, mitochondria receive less pyruvate-derived acetyl-CoA, potentially reducing oxidative glucose metabolism and promoting compensatory lactate production [16,18]. This may be useful during acute nutrient adaptation, but chronic PDK4 activation can contribute to metabolic inflexibility, redox imbalance, and inflammatory signaling [16]. The same PDK4 response may therefore be adaptive in one setting and maladaptive in another, depending on duration, tissue type, substrate availability, and inflammatory context. Therefore, PDK4 should not be viewed as uniformly harmful; its biological effect depends on whether it is transiently induced for metabolic adaptation or chronically activated in a stressed aging tissue.

### 3.2. PDK4-Associated Lactate Accumulation and Redox-Inflammatory Signaling

Recent work has placed PDK4 at the interface of senescence, lactate metabolism, and inflammatory amplification [16]. Dou and colleagues showed that senescent cells develop a PDK4-dependent hypercatabolic state characterized by lactate production, and that lactate can promote ROS production through NOX1 to support SASP activity [16]. This finding directly connects restricted mitochondrial pyruvate oxidation with inflammatory output. In this model, increased PDK4 activity restricts pyruvate oxidation, favors lactate accumulation, and allows lactate–NOX1 signaling to increase ROS-dependent NF-κB and SASP activity.

The PDK4-lactate-NOX1-ROS-SASP pathway should be presented as a strong emerging model rather than as a universally proven mechanism in every aging tissue. The most direct evidence comes from senescent stromal cells and cancer-associated contexts [16]. Additional inflammatory disease models support the broader idea that PDK4-driven lactate accumulation can contribute to tissue injury and inflammatory responses, but tissue-specific validation in aging remains incomplete [33,34]. This distinction matters because the strongest evidence is currently from senescent stromal and cancer-associated models, whereas direct aging-tissue evidence remains limited.

To avoid overinterpretation, PDK4-related evidence should be considered at three levels. First, direct mechanistic evidence supports a PDK4-dependent lactate–NOX1–ROS–SASP axis in senescent stromal and cancer-associated settings. Second, tissue injury and inflammatory disease models support a broader connection between PDK4 activity, altered pyruvate oxidation, redox stress, and inflammation. Third, in several aging tissues, including brain, kidney, and adipose tissue, PDK4 involvement remains a working hypothesis that requires cell-type-specific validation.

### 3.3. PDK4 and Mitochondrial Stress in Cellular Senescence

Senescent cells are metabolically active and often display elevated energy demand to sustain SASP production [16,35]. Their mitochondria are frequently dysfunctional, producing ROS and supporting inflammatory signaling rather than efficient bioenergetics. PDK4 may contribute to this phenotype by shifting pyruvate away from mitochondrial oxidation and toward lactate [16]. Lactate should not be viewed only as a glycolytic waste product; it can also act as a signaling metabolite [16]. In senescent tissues, PDK4-dependent lactate production may therefore act locally to reinforce SASP and non-cell-autonomously to activate neighboring cells.

This concept also links PDK4 to bystander inflammation. If lactate and ROS generated by senescent or metabolically stressed cells influence neighboring epithelial cells, fibroblasts, immune cells, or endothelial cells, then relatively small populations of senescent cells could amplify tissue-wide inflammation [36]. Such a model is consistent with the broader biology of SASP, in which senescent cells reshape tissue microenvironments through secreted cytokines, chemokines, proteases, extracellular vesicles, and metabolites. PDK4 may therefore contribute functionally to senescence-associated inflammation, rather than simply marking altered fuel use.

## 4. Mitochondria-Centered Inflammatory Pathways

### 4.1. NF-kappaB as a Redox-Sensitive Inflammatory Effector

NF-κB is one of the main transcriptional regulators of inflammatory gene expression. In the context of mitochondrial aging, NF-kappaB should be discussed not as an isolated pathway but as a downstream effector of redox imbalance, mitochondrial danger signaling, AGE-RAGE activation, cytokine stimulation, and impaired proteostasis [20,37]. mtROS can enhance NF-kappaB activation directly through redox-sensitive signaling intermediates and indirectly by promoting DNA damage, inflammasome activation, and senescence [4,37]. Once activated, NF-kappaB drives expression of IL-6, TNF-alpha, IL-1 pathway components, chemokines, cyclooxygenase-2, inducible nitric oxide synthase, and SASP mediators [20].

Inflammation also feeds back onto mitochondria. TNF-alpha, IL-1beta, interferons, and sustained NF-kappaB activity can impair mitochondrial respiration, alter mitochondrial dynamics, induce nitric oxide production, and increase oxidative stress [38]. This creates a bidirectional relationship in which mitochondrial stress activates NF-κB, while NF-κB-driven inflammation further impairs mitochondrial function.

### 4.2. NLRP3 Inflammasome Activation by Mitochondrial Damage

Among innate immune pathways, the NLRP3 inflammasome has one of the clearest links to mitochondrial damage in aging biology [3,39]. Multiple mitochondrial abnormalities can contribute to NLRP3 activation, including mtROS accumulation, oxidized mtDNA release, mitochondrial depolarization, lysosomal damage, potassium efflux, and impaired mitophagy [3,11,12,26]. After activation, NLRP3 promotes caspase-1-mediated maturation of IL-1beta and IL-18 and can trigger gasdermin D-dependent pyroptotic cell death [39]. In aging tissues, even low-level recurrent NLRP3 activation may maintain inflammation without overt infection [3].

The mitochondrial-NLRP3 connection is especially relevant to the brain, kidney, adipose tissue, and vasculature [3]. These tissues are highly vulnerable to mitochondrial dysfunction and are commonly affected by age-related inflammatory disease. Therapeutically, this suggests that inhibition of NLRP3 itself may be beneficial, but upstream restoration of mitochondrial quality control may be more durable because it removes the activating signal rather than only blocking one downstream node.

### 4.3. SASP as a Mitochondria-Dependent Secretory Program

The SASP is a major mechanism through which senescent cells influence tissue aging. Although it is often described as a transcriptional inflammatory program, SASP is also metabolically demanding and strongly influenced by mitochondrial status [24,35]. Mitochondrial dysfunction can promote DNA damage responses, ROS accumulation, cytosolic chromatin fragments, cGAS-STING activation, and NF-kappaB signaling, all of which support SASP expression [24,35,40]. Conversely, reducing mitochondrial dysfunction or improving mitophagy can attenuate SASP in experimental systems [28].

PDK4 introduces a fuel-selection component to this model. By reducing pyruvate oxidation and promoting lactate accumulation, PDK4 may help sustain the bioenergetic and redox conditions required for inflammatory secretory activity [16]. SASP is therefore better viewed as an active mitochondrial and metabolic output of senescent cells, not just as a marker of senescence.

### 4.4. AGE-RAGE Signaling as an Amplifier of Mitochondrial ROS

Advanced glycation end products accumulate during aging and metabolic disease through non-enzymatic glycation and oxidative modification of proteins and lipids [41]. Binding of AGEs to RAGE activates NF-kappaB, MAPK, JAK-STAT, and NADPH oxidase pathways, promoting inflammation and oxidative stress [41]. From a mitochondrial perspective, AGE-RAGE signaling is important because it amplifies ROS production, impairs endothelial mitochondrial function, and contributes to vascular stiffness, renal injury, and neuroinflammation [42]. Thus, AGE-RAGE should be viewed as an extrinsic metabolic stress pathway that feeds into mitochondrial redox dysfunction and inflammatory signaling.

## 5. Tissue-Specific Mitochondrial Meta-Inflammation

### 5.1. Skeletal Muscle

**Figure 3:**
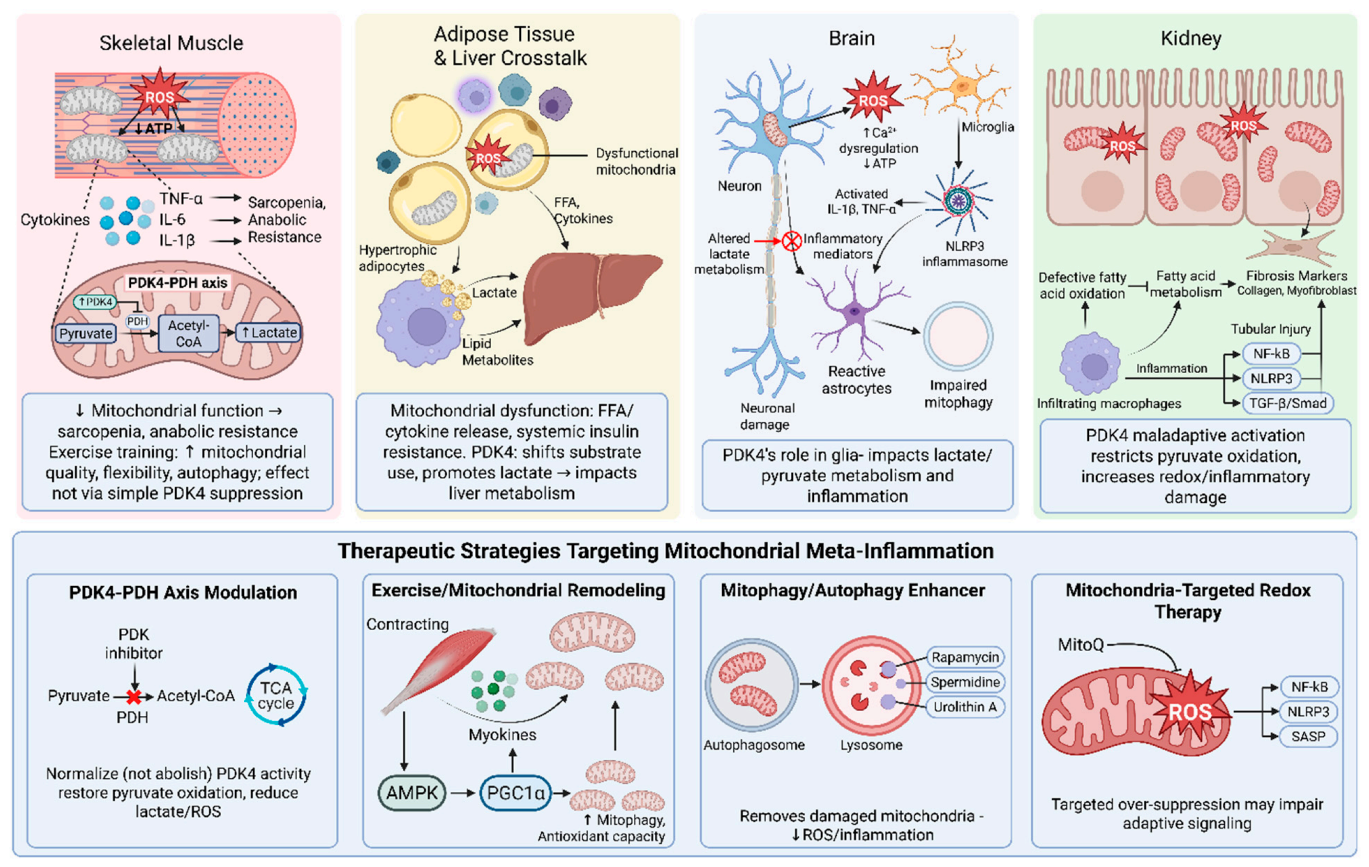
**Tissue-specific mitochondrial meta-inflammation and therapeutic strategies targeting the PDK4–mitochondrial axis.** Aging tissues show distinct patterns of mitochondrial inflammatory stress. In skeletal muscle, mitochondrial dysfunction, oxidative stress, inflammatory cytokines, and altered PDK4–PDH signaling contribute to impaired glucose oxidation, anabolic resistance, and sarcopenia, whereas exercise training improves mitochondrial quality, metabolic flexibility, autophagy, and inflammatory balance. In adipose tissue, hypertrophic adipocytes with reduced oxidative capacity release fatty acids, cytokines, lactate, and danger signals that recruit macrophages and promote adipose–liver inflammatory crosstalk. In the brain, neuronal mitochondrial dysfunction, impaired mitophagy, mtROS accumulation, calcium dysregulation, microglial activation, astrocyte reactivity, and NLRP3 inflammasome signaling contribute to neuroinflammatory injury; the PDK4-specific role in brain aging remains under validation, but PDK-mediated pyruvate/lactate handling may influence glial metabolism and inflammatory tone. In the kidney, proximal tubular mitochondrial dysfunction, defective fatty acid oxidation, macrophage infiltration, NF-κB/NLRP3 activation, and TGF-β/Smad signaling promote tubular injury and fibrosis; PDK4 may contribute to redox-inflammatory injury in renal stress contexts, although direct links to renal aging require further study. Therapeutic strategies include PDK4–PDH axis modulation, exercise-mediated mitochondrial remodeling, mitophagy/autophagy enhancement, and mitochondria-targeted redox therapy. These interventions aim to improve mitochondrial fuel use, remove damaged mitochondria, restore redox balance, and limit chronic inflammation. Arrows indicate tissue-specific metabolic and inflammatory links, and red ROS symbols indicate oxidative stress or redox-inflammatory signaling. Created with BioRender.com (BioRender, Toronto, ON, Canada; https://www.biorender.com/; accessed on 1 July 2026).

Skeletal muscle is highly dependent on mitochondrial oxidative metabolism, and age-associated decline in mitochondrial function contributes to reduced endurance, impaired glucose handling, anabolic resistance, and sarcopenia [43] (Figure 3). Inflammatory cytokines such as TNF-alpha, IL-6, and IL-1beta suppress anabolic signaling and promote proteolysis, while mitochondrial dysfunction reduces metabolic flexibility and increases oxidative stress [44]. In skeletal muscle, mitochondrial ROS and cytokine signaling can activate NF-κB-dependent inflammatory programs and promote catabolic pathways that impair protein synthesis and increase proteolysis [38,44]. NLRP3-related inflammatory signaling may also contribute to muscle wasting when mitochondrial damage and defective quality control persist [23,44]. These pathways connect mitochondrial stress with anabolic resistance, sarcopenia, and reduced metabolic flexibility during aging. PDK4 is particularly relevant in muscle because it regulates the balance between glucose oxidation and fatty acid utilization [45]. Acute exercise and fasting can induce PDK4 as part of physiological substrate switching, whereas chronic metabolic disease or aging-associated dysregulation may contribute to impaired glucose oxidation and insulin resistance [45].

Exercise should therefore be described carefully. It is not accurate to state universally that exercise suppresses PDK4. Acute endurance exercise often increases PDK4 expression, especially under conditions of glycogen depletion or fatty acid availability [46]. However, regular exercise training improves mitochondrial capacity, insulin sensitivity, autophagy, antioxidant defense, and inflammatory profiles. The anti-inflammatory benefit of exercise is therefore best attributed to improved mitochondrial quality and metabolic flexibility rather than simple suppression of PDK4 [46].

### 5.2. Adipose Tissue and Liver Crosstalk

Aging adipose tissue displays mitochondrial dysfunction, reduced adipocyte oxidative capacity, increased lipolysis, immune cell infiltration, and formation of crown-like structures around dying adipocytes [47]. Hypertrophic adipocytes release free fatty acids, cytokines, and danger signals that recruit and activate macrophages. Mitochondrial dysfunction in adipocytes and macrophages promotes ROS production and inflammatory polarization, linking local adipose stress to systemic insulin resistance [47]. In adipose tissue, mitochondrial ROS, lipid overload, and adipocyte stress can enhance NF-κB-dependent cytokine production and promote recruitment of inflammatory macrophages [47]. These macrophages further amplify local inflammation through TNF-α, IL-6, IL-1β, and chemokine signaling, while adipose-derived fatty acids and cytokines can propagate inflammatory stress to the liver [47,48]. This adipose–liver communication links local mitochondrial dysfunction with systemic insulin resistance and metabolic inflammation. Lactate and lipid-derived metabolites may also influence hepatic macrophages and hepatocytes, contributing to adipose–liver inflammatory crosstalk in metabolic syndrome [48].

PDK4 may participate in this adipose–liver axis by altering substrate selection and, in senescent or metabolically stressed stromal contexts, promoting lactate generation [16]. However, the tissue-specific role of PDK4 in aged adipose tissue requires more direct experimental validation.

### 5.3. Brain

The aging brain is highly vulnerable to mitochondrial dysfunction because neurons are post-mitotic, energetically demanding, and dependent on oxidative phosphorylation for synaptic transmission, axonal maintenance, ion homeostasis, and neurotransmitter cycling [49]. Mitochondrial impairment in neurons can reduce ATP availability, increase mtROS, disturb calcium buffering, and weaken synaptic resilience [49]. These changes are not restricted to neurons. They also influence astrocytes, microglia, and endothelial cells, creating a multicellular inflammatory environment in which mitochondrial stress and neuroimmune activation reinforce one another [50].

Neuron–glia metabolic coupling is particularly important in this context. Astrocytes support neuronal function by regulating extracellular glutamate, maintaining antioxidant defense, and providing metabolic substrates, including lactate, through the astrocyte–neuron lactate shuttle [51]. During aging or neurodegenerative stress, altered astrocytic metabolism may disturb this coupling, leading to impaired lactate handling, reduced neuronal metabolic support, and increased inflammatory signaling [51]. Because PDK enzymes regulate pyruvate entry into mitochondrial oxidation, PDK-mediated changes in astrocytic or microglial pyruvate/lactate metabolism could influence neuroinflammatory tone [18,52]. However, direct evidence for PDK4 as a causal regulator of brain aging remains limited, and this possibility should be tested in cell-type-specific aging models.

Microglia add another layer to mitochondrial meta-inflammation in the brain. Aging microglia often show altered immunometabolism, reduced mitochondrial fitness, exaggerated responses to inflammatory stimuli, and increased production of cytokines such as IL-1β and TNF-α [50]. Damaged mitochondria can promote microglial activation through mtROS, impaired mitophagy, and release of mitochondrial danger signals [9,11,13]. Cytosolic mtDNA may engage cGAS–STING signaling, whereas oxidized mtDNA, mitochondrial depolarization, and defective mitophagy can lower the threshold for NLRP3 inflammasome activation [10,11,12,26]. These pathways may contribute to persistent neuroinflammation, synaptic dysfunction, and neuronal injury during aging.

Thus, the brain provides an important example of how mitochondrial dysfunction can be translated into tissue-specific inflammatory damage. At present, PDK4 should be viewed as an emerging hypothesis in brain aging rather than an established driver. The stronger conclusion is that mitochondrial pyruvate/lactate handling, whether mediated by PDK4 or related PDK isoforms, may influence neuron–glia metabolic communication and glial inflammatory activation [18,51,52].

### 5.4. Kidney

The renal tubule, particularly the proximal tubular epithelium, is highly enriched in mitochondria and relies on oxidative metabolism for solute transport [53]. Aging kidneys exhibit tubular mitochondrial dysfunction, reduced fatty acid oxidation, increased oxidative stress, interstitial fibrosis, glomerulosclerosis, and declining filtration capacity [54]. Mitochondrial injury in tubular cells can activate NF-kappaB, NLRP3, TGF-beta/Smad, and profibrotic signaling [54]. These pathways are functionally connected: mtROS and mitochondrial danger signals can promote NF-κB and NLRP3 activation, whereas persistent epithelial stress can enhance TGF-β/Smad signaling and fibroblast activation [23,34,54]. In this way, mitochondrial damage links tubular inflammation with progressive fibrosis. Inflammatory macrophage infiltration and fibroblast activation then reinforce mitochondrial damage and fibrosis.

SGLT2 inhibitors provide an example of therapy that may reduce renal inflammation partly by improving metabolic stress, tubular workload, and mitochondrial function, beyond their glucose-lowering effects [55]. Although the precise mitochondrial mechanisms remain an active area of investigation, the kidney illustrates the broader principle that improving organelle metabolism can reduce inflammatory injury and fibrotic progression.

Direct evidence linking PDK4 to renal aging remains limited, but the PDK–PDH axis is relevant to kidney injury because tubular epithelial cells depend on mitochondrial substrate oxidation for transport and repair. In inflammatory or ischemic kidney stress, maladaptive PDK4 activation may restrict pyruvate oxidation, promote redox imbalance, and contribute to inflammatory injury. Indeed, inhibition of PDK4 has been reported to reduce oxidative stress and inflammation in renal ischemia–reperfusion injury models, suggesting that PDK4 may become important in kidney disease contexts where mitochondrial metabolism is disrupted [34]. Whether this mechanism contributes to renal fibroinflammation during aging remains unresolved. A summary of tissue-specific mitochondrial inflammatory features, proposed PDK4 involvement, and evidence strength is provided in Table 1.

## 6. Therapeutic Strategies Targeting Mitochondrial Meta-Inflammation

Therapeutic approaches can be grouped into two practical directions: restoring mitochondrial fuel flux and improving mitochondrial quality control, redox resilience, and stress adaptation. The PDK4–PDH axis is especially relevant because it directly controls whether pyruvate enters mitochondrial oxidation or is diverted toward lactate-linked inflammatory signaling [16,56]. These therapeutic approaches should be interpreted at different levels of evidence. Some interventions, such as exercise, have broad physiological and clinical support for improving mitochondrial health [21,22]. Others, including mitophagy enhancers, PDK inhibitors, NAD^+^ precursors, and mitochondria-targeted antioxidants, are supported by varying combinations of mechanistic rationale, preclinical data, and early or context-dependent clinical evidence [29,56,57,58,59]. Therefore, their relevance to human aging-associated meta-inflammation should be presented cautiously, with attention to tissue specificity, timing, dose, and patient selection.

### 6.1. Targeting the PDK4–PDH Axis to Restore Mitochondrial Pyruvate Oxidation

Modulating the PDK4–PDH axis directly targets mitochondrial pyruvate entry. PDK enzymes phosphorylate and inhibit the pyruvate dehydrogenase complex, thereby reducing conversion of pyruvate into acetyl-CoA and limiting glucose-derived carbon entry into the TCA cycle [56]. In aging or inflammatory stress, sustained PDK4 activation may restrict mitochondrial pyruvate oxidation, promote lactate accumulation, alter redox balance, and amplify ROS-sensitive inflammatory signaling [16].

Dichloroacetate is a classical PDK inhibitor that activates PDH flux by inhibiting PDK activity, demonstrating that pharmacological restoration of pyruvate oxidation is feasible [60]. However, chronic clinical use of dichloroacetate is limited by safety concerns, including peripheral neuropathy, emphasizing the need for more selective and context-specific strategies [61]. More selective PDK isoform inhibitors, tissue-targeted delivery systems, and combination approaches may provide safer ways to normalize maladaptive PDK4 activity without eliminating its physiological roles [56,60].

Complete PDK4 suppression is unlikely to be desirable, since PDK4 supports normal substrate switching during fasting, exercise, and increased fatty acid use. A more realistic goal is to normalize maladaptive PDK4 activity only in settings where chronic restriction of pyruvate oxidation drives lactate accumulation, redox stress, and inflammation [16,56].

### 6.2. Exercise and Mitochondrial Remodeling

Exercise is the most robust non-pharmacological intervention for improving mitochondrial function. It activates AMPK, calcium–calmodulin signaling, p38 MAPK, and PGC-1α-dependent mitochondrial biogenesis, while also enhancing mitophagy, antioxidant defense, insulin sensitivity, and metabolic flexibility [21,22]. These effects are systemic because contracting skeletal muscle releases myokines, improves glucose and lipid utilization, reduces visceral adiposity, and modulates immune-cell metabolism [21].

In relation to PDK4, exercise should be discussed carefully. Acute endurance exercise can increase PDK4 expression, particularly during glycogen depletion or increased fatty acid availability, reflecting physiological substrate switching rather than pathology. In contrast, regular exercise training improves mitochondrial capacity and metabolic flexibility, which may reduce the harmful consequences of chronic PDK4 dysregulation. Thus, exercise is better viewed as a way to improve mitochondrial adaptability, not simply to suppress PDK4.

### 6.3. Mitophagy and Autophagy Enhancers

Because defective mitochondrial clearance allows damaged mitochondria to persist as sources of mtROS, mtDNA, and inflammatory danger signals, enhancement of autophagy and mitophagy is an attractive therapeutic strategy. Rapamycin and rapalogs inhibit mTORC1 and can enhance autophagy, although long-term systemic mTOR inhibition may have context-dependent risks [57]. Spermidine promotes autophagy through mechanisms that include EP300 inhibition and has been linked to cardiometabolic and immune benefits in experimental and human observational settings [58].

Urolithin A is particularly relevant to a mitochondria-centered review because it activates mitophagy and improves muscle function in preclinical models [29]. These interventions do not directly target PDK4, but they may complement PDK4–PDH-axis modulation by removing damaged mitochondria and reducing the inflammatory consequences of mitochondrial stress.

### 6.4. NAD^+^ Metabolism and Sirtuin-Linked Mitochondrial Maintenance

NAD^+^ is essential for mitochondrial redox reactions and for the activity of sirtuins, PARPs, and other NAD^+^-dependent enzymes involved in DNA repair, metabolic regulation, stress responses, and cellular senescence [62]. NAD^+^ levels decline in aging tissues, potentially impairing mitochondrial function, genome maintenance, and inflammatory control [62].

NAD^+^ precursors such as nicotinamide riboside and nicotinamide mononucleotide can increase NAD-related metabolites in humans, but their clinical effects on physical function, inflammation, mitochondrial performance, and healthspan remain variable [63]. NAD^+^ repletion should therefore be discussed cautiously. It is mechanistically attractive and biologically active, but larger and longer studies are needed to determine whether it improves mitochondrial dysfunction or meta-inflammatory outcomes in aging.

### 6.5. Mitochondria-Targeted Redox Therapy

Conventional antioxidants have produced inconsistent clinical benefits, partly because ROS also serve physiological signaling functions and because untargeted antioxidants may not reach the most relevant subcellular compartments. Mitochondria-targeted redox approaches, such as MitoQ, are designed to accumulate within mitochondria and reduce pathological oxidative stress closer to its source [59].

These approaches are conceptually attractive for mitochondrial meta-inflammation because mtROS lies upstream of NF-κB activation, NLRP3 inflammasome priming, mitochondrial danger signaling, and SASP amplification. However, clinical translation requires careful patient selection, biomarker-guided dosing, and recognition that excessive ROS suppression may interfere with adaptive mitohormetic signaling. In a PDK4-centered model, mitochondrial redox therapies may be most effective when combined with strategies that restore pyruvate oxidation, improve mitophagy, and normalize metabolic flexibility.

## 7. Future Directions

Several questions now need to be answered through direct experimental testing. First, the field needs tissue- and cell-type-specific maps of mitochondrial dysfunction during aging, integrating respiration, metabolomics, mtDNA release, mitophagy flux, and inflammatory output. Second, PDK4 should be tested in defined aging-relevant cell types, including senescent fibroblasts, macrophages, adipocytes, skeletal muscle fibers, astrocytes, microglia, and renal tubular epithelial cells. Third, studies should distinguish adaptive transient PDK4 induction from maladaptive chronic PDK4 activation. Fourth, biomarkers such as lactate, PDH phosphorylation, NOX1 activity, mtROS, circulating mtDNA, IL-6, IL-1beta, and SASP panels should be integrated into human aging cohorts. Finally, therapeutic studies should test combinations that restore mitochondrial function, enhance mitophagy, normalize PDH flux, and reduce inflammatory output, rather than relying on single-pathway inhibition.

## 8. Conclusions

Mitochondrial dysfunction offers a practical way to understand why metabolic stress and chronic inflammation become linked during aging. Declining oxidative phosphorylation, excess mtROS, mtDNA release, impaired mitophagy, altered NAD^+^ metabolism, and disrupted pyruvate oxidation converge on NF-kappaB, NLRP3, cGAS-STING, and SASP programs. PDK4 is important in this network because it controls pyruvate entry into mitochondria and may influence lactate-linked ROS and SASP signaling. This PDK4-centered interpretation provides a distinct perspective by placing pyruvate oxidation control within the broader network of mitochondrial danger signaling, senescence, and immune-metabolic inflammation. The key message is that metabolism and inflammation should not be treated as separate aging processes. Mitochondrial metabolic failure can itself generate inflammatory signals that spread across cells and tissues. Interventions that improve mitochondrial function, including exercise, mitophagy activation, NAD^+^-linked metabolic support, mitochondria-targeted redox modulation, and calibrated PDK4–PDH-axis targeting, may therefore help reduce meta-inflammation and preserve healthspan.

## Figures and Tables

**Table 1:** Tissue-specific mitochondrial meta-inflammation and proposed PDK4 involvement.

Tissue/Context	Mitochondrial-Inflammatory Features	PDK4 Role	Evidence
Senescent stromal/cancer-associated cells	Lactate, ROS, NF-κB/SASP	Supports lactate–NOX1–ROS–SASP signaling	Direct
Skeletal muscle	Low oxidative capacity, metabolic inflexibility, catabolism	Regulates glucose–fatty acid switching	Moderate
Adipose–liver axis	Adipocyte mitochondrial stress, macrophages, FFA/cytokines	May promote lactate-linked inflammatory crosstalk	Emerging
Brain	Neuron–glia stress, mtROS, mitophagy defects, NLRP3/cGAS–STING	PDK4 role unclear; PDK-mediated pyruvate/lactate handling may affect glia	Limited
Kidney	Tubular mitochondrial dysfunction, defective FAO, fibrosis pathways	PDK4 inhibition reduces injury- related oxidative inflammation	Preclinical

## Data Availability

No new data were created or analyzed in this study. Data sharing is not applicable to this article.

## References

[1] López-Otín C., Blasco M.A., Partridge L., Serrano M., Kroemer G. (2023). Hallmarks of Aging: An Expanding Universe. Cell.

[2] Furman D., Campisi J., Verdin E., Carrera-Bastos P., Targ S., Franceschi C., Ferrucci L., Gilroy D.W., Fasano A., Miller G.W. (2019). Chronic Inflammation in the Etiology of Disease across the Life Span. Nat. Med..

[3] Xu X., Pang Y., Fan X. (2025). Mitochondria in Oxidative Stress, Inflammation and Aging: From Mechanisms to Therapeutic Advances. Signal Transduct. Target. Ther..

[4] Zong Y., Li H., Liao P., Chen L., Pan Y., Zheng Y., Zhang C., Liu D., Zheng M., Gao J. (2024). Mitochondrial Dysfunction: Mechanisms and Advances in Therapy. Signal Transduct. Target. Ther..

[5] Harrington J.S., Ryter S.W., Plataki M., Price D.R., Choi A.M.K. (2023). Mitochondria in Health, Disease, and Aging. Physiol. Rev..

[6] O’Neill L.A.J., Kishton R.J., Rathmell J. (2016). A Guide to Immunometabolism for Immunologists. Nat. Rev. Immunol..

[7] Yun J., Finkel T. (2014). Mitohormesis. Cell Metab..

[8] Min S.H., Kang G.M., Park J.W., Kim M.S. (2024). Beneficial Effects of Low-Grade Mitochondrial Stress on Metabolic Diseases and Aging. Yonsei Med. J..

[9] Lazarou M., Sliter D.A., Kane L.A., Sarraf S.A., Wang C., Burman J.L., Sideris D.P., Fogel A.I., Youle R.J. (2015). The Ubiquitin Kinase PINK1 Recruits Autophagy Receptors to Induce Mitophagy. Nature.

[10] West A.P., Khoury-Hanold W., Staron M., Tal M.C., Pineda C.M., Lang S.M., Bestwick M., Duguay B.A., Raimundo N., MacDuff D.A. (2015). Mitochondrial DNA Stress Primes the Antiviral Innate Immune Response. Nature.

[11] Jiménez-Loygorri J.I., Sorrentino T., Di Micco A., Villarejo-Zori B., Viedma-Poyatos Á., Zapata-Muñoz J., Benítez-Fernández R., Frutos-Lisón M.D., Tomás-Barberán F.A., Espín J.C. (2024). Mitophagy curtails cytosolic mtDNA-dependent activation of cGAS/STING inflammation during aging. Nat. Commun..

[12] Iyer S.S., He Q., Janczy J.R., Elliott E.I., Zhong Z., Olivier A.K., Sadler J.J., Knepper-Adrian V., Han R., Qiao L. (2013). Mitochondrial Cardiolipin Is Required for Nlrp3 Inflammasome Activation. Immunity.

[13] Palmer J.E., Wilson N., Son S.M., Obrocki P., Wrobel L., Rob M., Takla M., Korolchuk V.I., Rubinsztein D.C. (2025). Autophagy, Aging, and Age-Related Neurodegeneration. Neuron.

[14] Ferrucci L., Fabbri E. (2018). Inflammageing: Chronic Inflammation in Ageing, Cardiovascular Disease, and Frailty. Nat. Rev. Cardiol..

[15] Hotamisligil G.S. (2017). Inflammation, Metaflammation and Immunometabolic Disorders. Nature.

[16] Dou X., Lee S.L.Y., Lin W., Zou Y., Fu D., Xu Q., Jiang Z., Ren X., Zhang G., Wei X. (2023). PDK4-dependent hypercatabolism and lactate production of senescent cells promotes cancer malignancy. Nat. Metab..

[17] Zhang X., Gao Y., Zhang S., Wang Y., Pei X., Chen Y., Zhang J., Zhang Y., Du Y., Hao S. (2025). Mitochondrial Dysfunction in the Regulation of Aging and Aging-Related Diseases. Cell Commun. Signal..

[18] Patel M.S., Nemeria N.S., Furey W., Jordan F. (2014). The Pyruvate Dehydrogenase Complexes: Structure-Based Function and Regulation. J. Biol. Chem..

[19] Forrester S.J., Kikuchi D.S., Hernandes M.S., Xu Q., Griendling K.K. (2018). Reactive Oxygen Species in Metabolic and Inflammatory Signaling. Circ. Res..

[20] Capece D., Verzella D., Flati I., Arboretto P., Cornice J., Franzoso G. (2022). NF-ΚB: Blending Metabolism, Immunity, and Inflammation. Trends Immunol..

[21] Hawley J.A., Hargreaves M., Joyner M.J., Zierath J.R. (2014). Integrative Biology of Exercise. Cell.

[22] Memme J.M., Erlich A.T., Phukan G., Hood D.A. (2021). Exercise and Mitochondrial Health. J. Physiol..

[23] Gupta S., Cassel S.L., Sutterwala F.S., Dagvadorj J. (2025). Regulation of the NLRP3 Inflammasome by Autophagy and Mitophagy. Immunol. Rev..

[24] Wiley C.D., Velarde M.C., Lecot P., Liu S., Sarnoski E.A., Freund A., Shirakawa K., Lim H.W., Davis S.S., Ramanathan A. (2016). Mitochondrial Dysfunction Induces Senescence with a Distinct Secretory Phenotype. Cell Metab..

[25] Kim J., Kim H.S., Chung J.H. (2023). Molecular Mechanisms of Mitochondrial DNA Release and Activation of the CGAS-STING Pathway. Exp. Mol. Med..

[26] Shimada K., Crother T.R., Karlin J., Dagvadorj J., Chiba N., Chen S., Ramanujan V.K., Wolf A.J., Vergnes L., Ojcius D.M. (2012). Oxidized Mitochondrial DNA Activates the NLRP3 Inflammasome during Apoptosis. Immunity.

[27] Kane L.A., Lazarou M., Fogel A.I., Li Y., Yamano K., Sarraf S.A., Banerjee S., Youle R.J. (2014). PINK1 Phosphorylates Ubiquitin to Activate Parkin E3 Ubiquitin Ligase Activity. J. Cell Biol..

[28] Korolchuk V.I., Miwa S., Carroll B., von Zglinicki T. (2017). Mitochondria in Cell Senescence: Is Mitophagy the Weakest Link?. EBioMedicine.

[29] Ryu D., Mouchiroud L., Andreux P.A., Katsyuba E., Moullan N., Nicolet-Dit-Félix A.A., Williams E.G., Jha P., Lo Sasso G., Huzard D. (2016). Urolithin A Induces Mitophagy and Prolongs Lifespan in *C. elegans* and Increases Muscle Function in Rodents. Nat. Med..

[30] Jeong J.Y., Jeoung N.H., Park K.G., Lee I.K. (2012). Transcriptional Regulation of Pyruvate Dehydrogenase Kinase. Diabetes Metab. J..

[31] Connaughton S., Chowdhury F., Attia R.R., Song S., Zhang Y., Elam M.B., Cook G.A., Park E.A. (2010). Regulation of Pyruvate Dehydrogenase Kinase Isoform 4 (PDK4) Gene Expression by Glucocorticoids and Insulin. Mol. Cell. Endocrinol..

[32] Yamaguchi S., Moseley A.C., Valdes P.A., Stromsdorfer K.L., Franczyk M.P., Okunade A.L., Patterson B.W., Klein S., Yoshino J. (2018). Diurnal Variation in PDK4 Expression Is Associated with Plasma Free Fatty Acid Availability in People. J. Clin. Endocrinol. Metab..

[33] Lee H., Jeon J.H., Lee Y.J., Kim M.J., Kwon W.H., Chanda D., Thoudam T., Pagire H.S., Pagire S.H., Ahn J.H. (2023). Inhibition of Pyruvate Dehydrogenase Kinase 4 in CD4+ T Cells Ameliorates Intestinal Inflammation. Cell. Mol. Gastroenterol. Hepatol..

[34] Khang A.R., Kim D.H., Kim M.J., Oh C.J., Jeon J.H., Choi S.H., Lee I.K. (2024). Reducing Oxidative Stress and Inflammation by Pyruvate Dehydrogenase Kinase 4 Inhibition Is Important in Prevention of Renal Ischemia-Reperfusion Injury in Diabetic Mice. Diabetes Metab. J..

[35] Martini H., Passos J.F. (2023). Cellular Senescence: All Roads Lead to Mitochondria. FEBS J..

[36] Borghesan M., Fafián-Labora J., Eleftheriadou O., Carpintero-Fernández P., Paez-Ribes M., Vizcay-Barrena G., Swisa A., Kolodkin-Gal D., Ximénez-Embún P., Lowe R. (2019). Small Extracellular Vesicles Are Key Regulators of Non-Cell Autonomous Intercellular Communication in Senescence via the Interferon Protein IFITM3. Cell Rep..

[37] Morgan M.J., Liu Z.G. (2011). Crosstalk of Reactive Oxygen Species and NF-ΚB Signaling. Cell Res..

[38] Nisr R.B., Shah D.S., Ganley I.G., Hundal H.S. (2019). Proinflammatory NFkB Signalling Promotes Mitochondrial Dysfunction in Skeletal Muscle in Response to Cellular Fuel Overloading. Cell. Mol. Life Sci..

[39] Swanson K.V., Deng M., Ting J.P.Y. (2019). The NLRP3 Inflammasome: Molecular Activation and Regulation to Therapeutics. Nat. Rev. Immunol..

[40] Glück S., Guey B., Gulen M.F., Wolter K., Kang T.W., Schmacke N.A., Bridgeman A., Rehwinkel J., Zender L., Ablasser A. (2017). Innate Immune Sensing of Cytosolic Chromatin Fragments through CGAS Promotes Senescence. Nat. Cell Biol..

[41] Rungratanawanich W., Qu Y., Wang X., Essa M.M., Song B.J. (2021). Advanced Glycation End Products (AGEs) and Other Adducts in Aging-Related Diseases and Alcohol-Mediated Tissue Injury. Exp. Mol. Med..

[42] Senatus L.M., Schmidt A.M. (2017). The AGE-RAGE Axis: Implications for Age-Associated Arterial Diseases. Front. Genet..

[43] Kim M.J., Sinam I.S., Siddique Z., Jeon J.H., Lee I.K. (2023). The Link between Mitochondrial Dysfunction and Sarcopenia: An Update Focusing on the Role of Pyruvate Dehydrogenase Kinase 4. Diabetes Metab. J..

[44] Ji Y., Li M., Chang M., Liu R., Qiu J., Wang K., Deng C., Shen Y., Zhu J., Wang W. (2022). Inflammation: Roles in Skeletal Muscle Atrophy. Antioxidants.

[45] Pin F., Novinger L.J., Huot J.R., Harris R.A., Couch M.E., O’Connell T.M., Bonetto A. (2019). PDK4 Drives Metabolic Alterations and Muscle Atrophy in Cancer Cachexia. FASEB J..

[46] Zhu Y., Zhou X., Zhu A., Xiong S., Xie J., Bai Z. (2023). Advances in Exercise to Alleviate Sarcopenia in Older Adults by Improving Mitochondrial Dysfunction. Front. Physiol..

[47] Woo C.Y., Jang J.E., Lee S.E., Koh E.H., Lee K.U. (2019). Mitochondrial Dysfunction in Adipocytes as a Primary Cause of Adipose Tissue Inflammation. Diabetes Metab. J..

[48] Rosso C., Kazankov K., Younes R., Esmaili S., Marietti M., Sacco M., Carli F., Gaggini M., Salomone F., Møller H.J. (2019). Crosstalk between Adipose Tissue Insulin Resistance and Liver Macrophages in Non-Alcoholic Fatty Liver Disease. J. Hepatol..

[49] Yang S., Park J.H., Lu H.C. (2023). Axonal Energy Metabolism, and the Effects in Aging and Neurodegenerative Diseases. Mol. Neurodegener..

[50] Kwon H.S., Koh S.H. (2020). Neuroinflammation in Neurodegenerative Disorders: The Roles of Microglia and Astrocytes. Transl. Neurodegener..

[51] Newington J.T., Harris R.A., Cumming R.C. (2013). Reevaluating Metabolism in Alzheimer’s Disease from the Perspective of the Astrocyte-Neuron Lactate Shuttle Model. J. Neurodegener. Dis..

[52] Rahman M.H., Bhusal A., Kim J.H., Jha M.K., Song G.J., Go Y., Jang I.S., Lee I.K., Suk K. (2020). Astrocytic Pyruvate Dehydrogenase Kinase-2 Is Involved in Hypothalamic Inflammation in Mouse Models of Diabetes. Nat. Commun..

[53] Hoogstraten C.A., Hoenderop J.G., De Baaij J.H.F. (2026). Mitochondrial Dysfunction in Kidney Tubulopathies. Annu. Rev. Physiol..

[54] Fontecha-Barriuso M., Lopez-Diaz A.M., Guerrero-Mauvecin J., Miguel V., Ramos A.M., Sanchez-Niño M.D., Ruiz-Ortega M., Ortiz A., Sanz A.B. (2022). Tubular Mitochondrial Dysfunction, Oxidative Stress, and Progression of Chronic Kidney Disease. Antioxidants.

[55] Sen T., Heerspink H.J.L. (2021). A Kidney Perspective on the Mechanism of Action of Sodium Glucose Co-Transporter 2 Inhibitors. Cell Metab..

[56] Schoenmann N., Tannenbaum N., Hodgeman R.M., Raju R.P. (2023). Regulating Mitochondrial Metabolism by Targeting Pyruvate Dehydrogenase with Dichloroacetate, a Metabolic Messenger. Biochim. Biophys. Acta Mol. Basis Dis..

[57] Lee D.J.W., Hodzic Kuerec A., Maier A.B. (2024). Targeting Ageing with Rapamycin and Its Derivatives in Humans: A Systematic Review. Lancet Healthy Longev..

[58] Pietrocola F., Lachkar S., Enot D.P., Niso-Santano M., Bravo-San Pedro J.M., Sica V., Izzo V., Maiuri M.C., Madeo F., Mariño G. (2015). Spermidine Induces Autophagy by Inhibiting the Acetyltransferase EP300. Cell Death Differ..

[59] Rossman M.J., Santos-Parker J.R., Steward C.A.C., Bispham N.Z., Cuevas L.M., Rosenberg H.L., Woodward K.A., Chonchol M., Gioscia-Ryan R.A., Murphy M.P. (2018). Chronic Supplementation with a Mitochondrial Antioxidant (MitoQ) Improves Vascular Function in Healthy Older Adults. Hypertension.

[60] James M.O., Jahn S.C., Zhong G., Smeltz M.G., Hu Z., Stacpoole P.W. (2017). Therapeutic Applications of Dichloroacetate and the Role of Glutathione Transferase Zeta-1. Pharmacol. Ther..

[61] Kaufmann P., Engelstad K., Wei Y., Jhung S., Sano M.C., Shungu D.C., Millar W.S., Hong X., Gooch C.L., Mao X. (2006). Dichloroacetate Causes Toxic Neuropathy in MELAS: A Randomized, Controlled Clinical Trial. Neurology.

[62] Covarrubias A.J., Perrone R., Grozio A., Verdin E. (2021). NAD+ Metabolism and Its Roles in Cellular Processes during Ageing. Nat. Rev. Mol. Cell Biol..

[63] Damgaard M.V., Treebak J.T. (2023). What Is Really Known about the Effects of Nicotinamide Riboside Supplementation in Humans. Sci. Adv..

